# Supplementary material for: Maxent estimation of aquatic Escherichia coli stream impairment
Source: PeerJ. 2018 Sep 13;6:e5610. doi: 10.7717/peerj.5610 (PMC6139247; doi:10.7717/peerj.5610)
Supplement: Figure S1 — Red line represents the logistic threshold, and dotted lines represent the 95% CI for the parameters estimated. Action function for (A) alkalinity, (B) BOD, (C) conductivity, (D) dissolved oxygen, (E) hardness, (F) NO3 , (G) PO4, and (H) water temperature are shown. [file peerj-06-5610-s001.pdf]

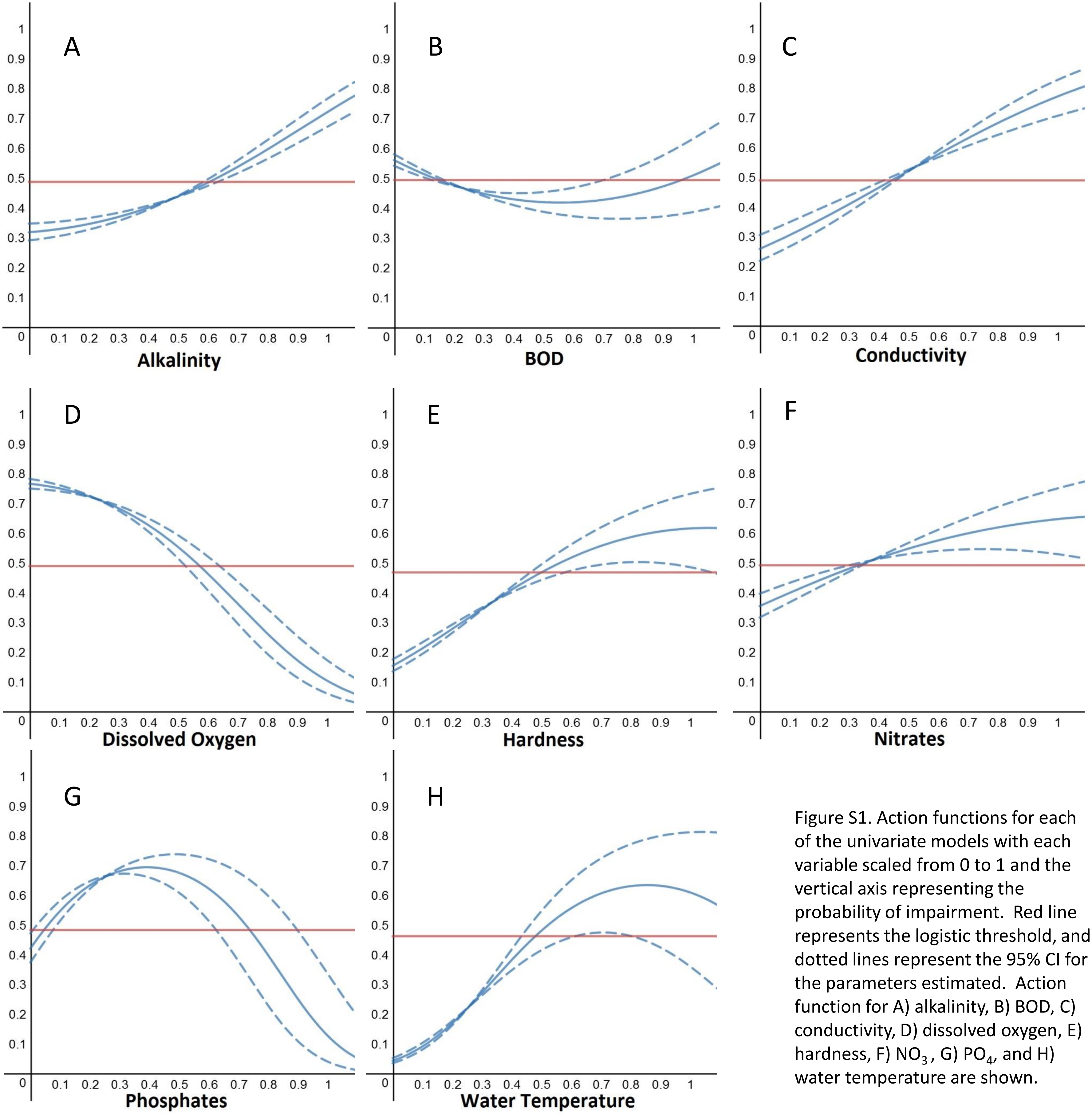

Figure S1. Action functions for each of the univariate models with each variable scaled from 0 to 1 and the vertical axis representing the probability of impairment. Red line represents the logistic threshold, and dotted lines represent the 95% CI for the parameters estimated. Action function for A) alkalinity, B) BOD, C) conductivity, D) dissolved oxygen, E) hardness, F) NO_3 , G) PO_4, and H) water temperature are shown.
