# Supplementary material for: Maxent estimation of aquatic Escherichia coli stream impairment
Source: PeerJ. 2018 Sep 13;6:e5610. doi: 10.7717/peerj.5610 (PMC6139247; doi:10.7717/peerj.5610)
Supplement: Figure S2 — This represents the mean probability of 100 bootstrapped runs. Rows are oriented by month of sampling, while columns represent each sampling site. [file peerj-06-5610-s002.pdf]

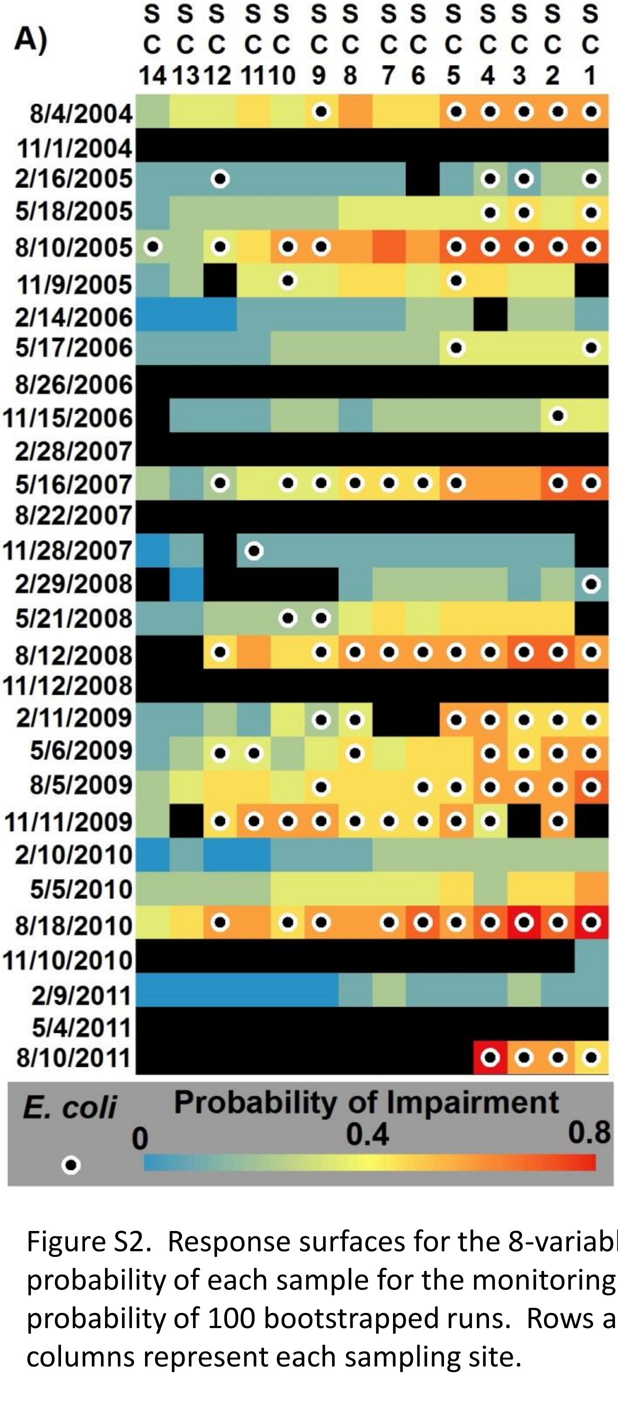

Figure S2. Response surfaces for the 8-variable (A) and 5-variable model (B) showing the probability of each sample for the monitoring program. This represents the mean probability of 100 bootstrapped runs. Rows are oriented by month of sampling, while columns represent each sampling site.
