# Supplementary material for: Maxent estimation of aquatic Escherichia coli stream impairment
Source: PeerJ. 2018 Sep 13;6:e5610. doi: 10.7717/peerj.5610 (PMC6139247; doi:10.7717/peerj.5610)
Supplement: Table S1 [file peerj-06-5610-s003.pdf]

Table S1. Summary Statistics of data used in univariate and multivariate Maxent models of *Escherichia coli* impairment.

	Mean	SD	Minimum	Maximum
Alkalinity	99.62	52.25	4.00	210.00
BOD	1.66	1.42	0.02	6.43
Conductivity	218.98	123.06	11.00	676.00
Discharge	0.77	2.74	0.00	32.75
Dissolved Oxygen	10.19	1.96	0.79	15.90
Hardness	126.15	57.03	7.30	256.70
Nitrates	1.44	0.89	0.00	5.37
pH	7.56	0.45	5.25	8.74
Phosphates	0.36	0.72	0.00	10.04
Water Temperature	12.39	4.40	1.30	24.50
