# Supplementary material for: Maxent estimation of aquatic Escherichia coli stream impairment
Source: PeerJ. 2018 Sep 13;6:e5610. doi: 10.7717/peerj.5610 (PMC6139247; doi:10.7717/peerj.5610)
Supplement: Table S2 [file peerj-06-5610-s004.pdf]

Table S2. Classification performance for all models run based on maximum test sensitivity and specificity as the logistic threshold (decision boundary)

**Variables**	**Logistic Threshold**	**TN**	**FN**	**FP**	**TP**	**χ^2**
Alk	0.488	140	44	44	51	24.61
BOD	0.495	110	36	74	59	12.04
Cond	0.490	129	41	55	54	19.05
DO	0.493	128	34	56	61	29.30
Flow	0.482	155	72	29	23	2.86
Hardness	0.469	102	30	82	65	14.34
Nitrates	0.493	134	52	50	43	9.16
pH	0.491	104	44	80	51	2.63
Phosphates	0.484	138	55	46	40	8.54
Water Temp	0.463	118	31	66	64	24.58
8 variables	0.383	144	20	40	75	82.94
4 variables	0.424	152	30	32	65	70.52
5 variables	0.430	151	29	33	66	71.26
