# Supplementary material for: Maxent estimation of aquatic Escherichia coli stream impairment
Source: PeerJ. 2018 Sep 13;6:e5610. doi: 10.7717/peerj.5610 (PMC6139247; doi:10.7717/peerj.5610)
Supplement: Table S3 [file peerj-06-5610-s005.pdf]

Table S3. Probability of Impairment and associated 95% confidence intervals for the total creek, each site, and each month that was sampled.

	8-variable Mean Probability (95% CI)	5-variable Mean Probability (95% CI)	4-variable Mean Probability (95% CI)
**Sinking Creek**	**0.338 (0.319, 0.358)**	**0.353 (0.334, 0.373)**	**0.359 (0.340, 0.378)**
**SC1**	**0.427 (0.343, 0.512)**	**0.456 (0.379, 0.534)**	**0.455 (0.376, 0.535)**
**SC2**	**0.434 (0.364, 0.503)**	**0.453 (0.384, 0.522)**	**0.456 (0.384, 0.527)**
**SC3**	**0.410 (0.334, 0.485)**	**0.430 (0.355, 0.505)**	**0.434 (0.359, 0.508)**
**SC4**	**0.409 (0.332, 0.486)**	**0.418 (0.351, 0.508)**	**0.430 (0.349, 0.510)**
**SC5**	**0.403 (0.337, 0.470)**	**0.418 (0.353, 0.483)**	**0.413 (0.348, 0.477)**
**SC6**	**0.373 (0.314,0.433)**	**0.385 (0.326, 0.444)**	**0.386 (0.327,0.445)**
**SC7**	**0.359 (0.295,0.423)**	**0.372 (0.308, 0.435)**	**0.371 (0.307, 0.434)**
**SC8**	**0.336 (0.269,0.403)**	**0.351 (0.285, 0.418)**	**0.360 (0.295, 0.425)**
**SC9**	**0.317 (0.242,0.391)**	**0.331 (0.255, 0.406)**	**0.343 (0.271, 0.416)**
**SC10**	**0.289 (0.232,0.347)**	**0.305 (0.247, 0.364)**	**0.315 (0.258, 0.372)**
**SC11**	**0.292 (0.221, 0.363)**	**0.303 (0.233, 0.373)**	**0.316 (0.249, 0.382)**
**SC12**	**0.276 (0.202, 0.350)**	**0.288 (0.214, 0.362)**	**0.303 (0.233, 0.374)**
**SC13**	**0.189 (0.140, 0.237)**	**0.195 (0.146, 0.245)**	**0.211 (0.167, 0.255)**
**SC14**	**0.176 (0.132, 0.221)**	**0.184 (0.139, 0.230)**	**0.187 (0.148, 0.226)**

**February**	**0.225**<br>**(0.223, 0.228)**	**0.211**<br>**(0.209, 0.214)**	**0.191**<br>**(0.188, 0.194)**
**May**	**0.353**<br>**(0.351, 0.356)**	**0.352**<br>**(0.349, 0.355)**	**0.344**<br>**(0.342, 0.347)**
**August**	**0.535**<br>**(0.531, 0.538)**	**0.529**<br>**(0.526, 0.532)**	**0.511**<br>**(0.508, 0.514)**
**November**	**0.317**<br>**(0.312, 0.323)**	**0.315**<br>**(0.309, 0.321)**	**0.299**<br>**(0.294, 0.305)**
